# Patient Empowerment Among Children and Adolescents with Inflammatory Bowel Disease (IBD) and Parents of IBD Patients—Use of Counseling Services and Lack of Knowledge About Transition

**DOI:** 10.3390/children12050620

**Published:** 2025-05-10

**Authors:** Kalina Kaul, Stefan Schumann, Jakob Felder, Jan Däbritz, Jan de Laffolie

**Affiliations:** 1 Department of General Pediatrics and Neonatology, University Children’s Hospital, University Giessen, 35392 Giessen, Hesse, Germany; kalina.kaul@paediat.med.uni-giessen.de (K.K.); jakob.felder@paediat.med.uni-giessen.de (J.F.); jan.delaffolie@paediat.med.uni-giessen.de (J.d.L.); 2 Clinic for Pediatrics and Adolescent Medicine, DRK-Kinderklinik Siegen, 57072 Siegen, North Rhine-Westphalia, Germany; 3 Department of Pediatrics, Klinikum Westbrandenburg, 14467 Potsdam, Brandenburg, Germany; jan.daebritz@klinikumwb.de; 4 Institute for Clinical Research and Systems Medicine, Health and Medical University (HMU), 14467 Potsdam, Brandenburg, Germany; 5 German Centre for Child and Adolescent Health (DZKJ), Site Greifswald/Rostock, 17475 Greifswald, Mecklenburg-Western Pomerania, Germany

**Keywords:** pediatric inflammatory bowel disease, patient empowerment, survey, parents, Crohn’s disease, ulcerative colitis, chronically ill children, counseling services, transition

## Abstract

**Background**: Children and adolescents with pediatric inflammatory bowel diseases (PIBD) face significant challenges, including emotional stress, social isolation, and interrupted education due to symptoms. Effective counseling and education empower these young patients and their families to actively participate in healthcare. This paper investigates the IBD needs analysis (CEDNA), focusing on counseling and transition services. **Methods:** The Study Group distributed questionnaires to PIBD patients and the parents of children and adolescents with PIBD across Germany, with all responses provided anonymously. We conducted a subgroup analysis based on patient age and time since diagnosis, as well as aspects of regional distribution and city size. Parents’ responses were analyzed by corresponding age groups to facilitate comparison with the patients’ responses. **Results:** From October 2021 to April 2022, 1158 questionnaires (patients 38.9%, *n* = 450; parents 61.1%, *n* = 708) were completed. In the group of 16–17-year-old patients, only 14.1% (*n* = 239) feel well informed about transition programs (parents 6.7% of *n* = 360). Depending on the disease duration, 2.1% to 6.9% of the patients surveyed (*n* = 292) feel well informed about PIBD (parents 3.3% to 7.5%, *n* = 361). Nutritional counseling is the most requested support service (patients 49.2%, *n* = 382; parents: service used for their children 41.9%, *n* = 578; parents: service used for themselves 46.1%, *n* = 575). **Conclusions:** PIBD patients, especially aged 12–17, lack knowledge and preparation for transition to adult care. While general PIBD management awareness is fair, targeted educational efforts are necessary. Trustworthy information sources and early, tailored counseling services could enhance transition experiences and improve long-term disease management and patient outcomes.

## 1. Introduction

Affected children and adolescents with pediatric inflammatory bowel diseases (PIBD) must cope with challenging symptoms such as abdominal pain, diarrhea, weight loss and fatigue, which can have a significant impact on their daily lives [1]. The burden of these symptoms often affects their ability to participate in regular activities, attend school regularly and engage with peers, which can lead to social isolation or difficulties with academic performance [2]. In addition, the unpredictability of the disease can cause emotional stress and anxiety, further complicating the normal developmental processes that adolescents go through during this important stage of life [3,4]. The goal of treatment for IBD is, among other things, to enable the patient to live as normally as possible. For successful long-term treatment, it is essential that patients and their families are well informed about the disease and receive the support they need to cope with the associated challenges [5].

Patient empowerment is the process by which patients are enabled to actively participate in their healthcare and make self-determined decisions [6]. For adolescents with PIBD, this can mean learning to better understand their symptoms, know their treatment options and interactively advocate for their health [7]. By strengthening their self-confidence and self-management skills, they can better cope with the challenges of their disease and improve their quality of life [8]. Effective counseling services and a structured transition from pediatric to adult healthcare are two important aspects in this regard [9,10].

Counseling services can provide a platform where young patients and their parents can voice their concerns and fears in a safe and confidential environment. Professionals who specialize in the needs of IBD patients can provide valuable information about the disease, explain treatment options and develop strategies for managing symptoms and side effects. This helps young patients to develop a better understanding of their condition and become proactive participants in their own healthcare [9]. Another important aspect is the education of families [11]. Counseling services also offer support to parents and siblings, who are often affected by the challenges associated with a family member’s illness. Through workshops and information events, families can learn how they can best help and support their affected relatives.

The transition from childhood to adolescence and finally to adulthood plays a crucial role in empowering chronically ill children and adolescents [12,13,14]. For children and adolescents with IBD such as Crohn’s disease or ulcerative colitis, this phase is particularly challenging [1]. The medical, psychological and social dimensions of the transition are particularly complex in these cases and require careful attention [15]. Medical care is a key component of the transition process for children and adolescents with IBD [16]. Many young patients must learn to manage their disease independently. This includes understanding their disease, taking medications correctly and being able to independently organize medical appointments. Managing their own health can be a significant challenge for young people, especially when they frequently experience symptoms. Education about the disease and training in self-management are therefore essential to provide children and young people with the necessary knowledge [17]. Transition of children and adolescents with IBD requires a holistic approach that combines medical, psychosocial and educational aspects [18]. This is the best way to ensure that young people are not only well cared for medically, but can also enter adult life emotionally strengthened and well-integrated [15]. Specific knowledge gaps may not only be age-specific. Regional differences and trust in transition programs can also be important.

In this paper, the results of the representative IBD needs analysis (CEDNA) are evaluated in detail, with a focus on counseling services and transition for young patients with IBD and affected parents. In particular, this paper examines the knowledge gaps, regional differences and trust in counseling services among PIBD patients and parents in Germany. The overall study design and its primary outcomes were published in 2023 [19]. One of the main objectives of the study was to investigate how well adolescents and their families cope with IBD. Here, the knowledge of patients and parents about counseling services and transition medicine was analyzed, taking into account both age and time since diagnosis. The findings should help in the development of future services tailored to specific age groups or other patient groups.

## 2. Methods

### 2.1. Data Basis

The data presented in this article are derived from the CEDNA survey, which originated as part of the IBD Children’s and Adolescents’ Quality of Care Improvement Network (CED-KQN) project. The project aimed to enhance the care provided to children and adolescents with IBD by analyzing various aspects of health services research, particularly in the contexts of eHealth and Big Data. The term “CEDNA” stands for the “IBD Needs Assessment” in German. A comprehensive description of the CEDNA survey’s methodology has already been published before [20]. The results from this survey contribute to the ongoing efforts of the CED-KQN project, aiming to identify areas for improvement in the quality of care for children and adolescents with IBD.

In brief, the survey involved two distinct versions of a questionnaire, which were distributed across Germany between October 2021 and April 2022. One questionnaire was designed for adolescent patients with IBD, aged 12 to 17 years, and included 28 questions. The other version, comprising 41 questions, was directed at parents of children and adolescents with IBD, from birth up to 17 years of age. Each questionnaire was accompanied by a patient information sheet to ensure participants were fully informed about the survey. The two versions were tailored to capture the unique needs and experiences of both patients and parents, providing valuable insights into the care and support required for managing IBD in young people [21].

Parents were asked to specify the age of their children in order to compare the parents’ responses with those of the patients. This allowed the researchers to analyze the parents’ perspectives in relation to the same age group of adolescents. The anonymous questionnaires could be completed both on paper and online.

The main aim of the questions was to identify the specific challenges of target groups with IBD. In addition to health-related questions, socio-demographic data such as age, gender, place of residence and family environment were also collected. Subgroup analyses examined how factors such as age and time since diagnosis influenced coping mechanisms and information needs.

The study used two main methods of data collection: online and paper questionnaires. Online responses were collected using the LimeSurvey tool (LimeSurvey GmbH, Hamburg, Germany; https://www.limesurvey.org/de (accessed on 5 May 2025)) and imported into Excel (Office 365, Microsoft Corporation, Redmond, WA, USA), while responses from paper questionnaires were entered manually. The data were analyzed at the study center of the University Children’s Hospital, Department of General Pediatrics and Neonatology in Giessen, Germany. Data were imported into SAS (version 9.4) for statistical analysis.

### 2.2. Presentation of Results

In this study, the terms “patients”, “adolescents”, or “young people” specifically refer to individuals aged 12 to 17 who participated in the survey. The term “parents” encompasses all parents who completed the questionnaire on behalf of their children, who were up to 17 years old. These are parents who completed the questionnaire for their affected children, regardless of whether their own child completed the questionnaire. To ensure the accuracy of the data, responses from participants who either skipped questions or provided incorrect responses (e.g., selecting multiple answers when only one was allowed) were excluded from the analysis. For the analysis of different subgroups, only those responses where participants fully answered the relevant questions and provided complete information on both age and the time since diagnosis were included.

We summarized the place of residence of the respondents as follows: northern federal states: Hamburg, Bremen, Lower Saxony, Schleswig-Holstein; eastern federal states: Berlin, Thuringia, Saxony, Saxony-Anhalt, Mecklenburg-Western Pomerania, Brandenburg; southern federal states: Bavaria, Baden-Württemberg; western federal states: North Rhine-Westphalia, Hesse, Saarland, Rhineland-Palatinate.

## 3. Results

### 3.1. General Information About the Participants of the Survey

The CEDNA survey, conducted between October 2021 and April 2022, distributed a total of 2810 questionnaires and received 1158 completed responses. Of these, 61.1% (*n* = 708) were submitted by parents on behalf of their children, while 38.9% (*n* = 450) were completed by the patients themselves. A detailed summary of the sociodemographic characteristics, as well as information on the types and progression of the diseases affecting the patients, has already been published before [20].

### 3.2. Level of Knowledge of Patients and Parents of Patients on IBD Topics

**Figure 1:**
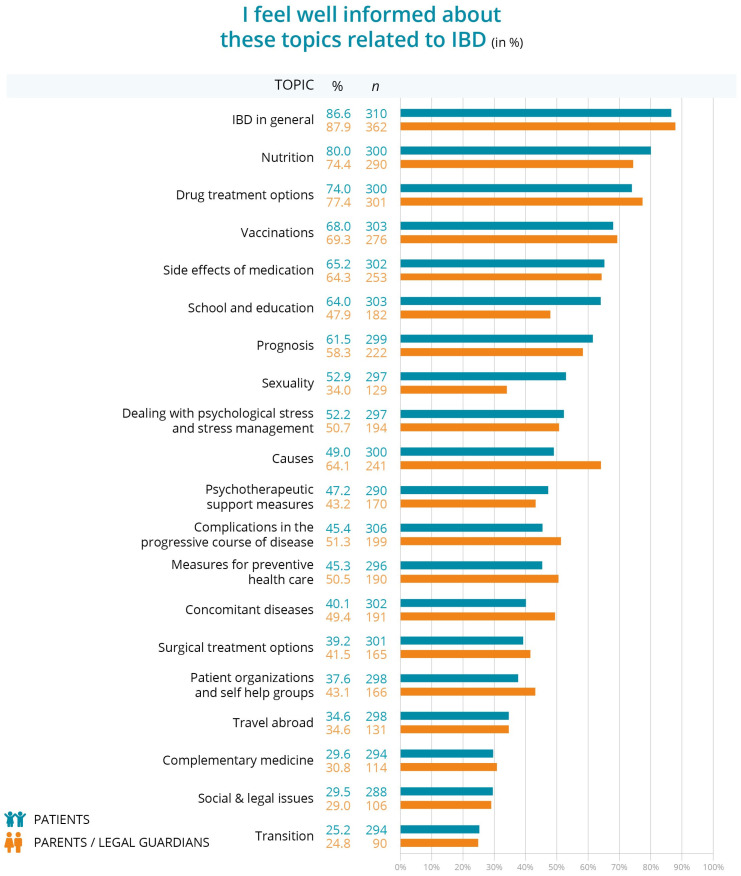
Level of knowledge of patients and parents of patients on various IBD topics.

Patients and parents of patients were asked which IBD-related topics they felt knowledgeable about. Both groups reported feeling most educated about general IBD aspects (patients 86.6%, *n* = 310; parents 87.9%, *n* = 362), nutrition (patients 80.0%, *n* = 300; parents 74.4%, *n* = 290), and available drug treatments (patients 74.0%, *n* = 300; parents 77.4%, *n* = 301) (Figure 1). The area they felt least informed about was the transition process, with only a quarter of patients (25.2%, *n* = 294) and a quarter of parents (24.8%, *n* = 90) feeling adequately informed about this topic. The highest information needs of patients were on the topics of causes for IBD (60.7%, *n* = 288), transition (58.5%, *n* = 283) and social law issues (52.6%, *n* = 272) and those of parents were on complementary medicine (23.5%, *n* = 310), transition (22.4%, *n* = 307) and on social law issues (22.2%, *n* = 310) [21].

Regarding the expressed desire for more information about the transition to adult care specifically, 12.1% of patients aged 12–13 years (*n* = 214) wanted to learn more about transition, increasing to 16.8% of patients aged 14–15 years (*n* = 214) and 31.8% of patients aged 16–17 years (*n* = 214). When considering the time since diagnosis, 6.7% of patients diagnosed for less than one year (*n* = 283) wanted more information on transition, compared to 17.3% of patients diagnosed for 1–2 years (*n* = 283), 13.4% for 3–4 years (*n* = 283), 8.1% for 5–6 years (*n* = 283), and 13.1% of patients diagnosed for more than 6 years (*n* = 283) [21].

All topics were divided into age and time since diagnosis subgroups for both the patients who participated directly and the children of the parents who participated on their behalf. Among these groups, the 16–17-year-olds generally felt the most knowledgeable on almost every topic, particularly regarding general IBD knowledge, nutrition, and drug treatment options. Of this age group, 14.1% reported feeling proficient about the transition process. The 12–13-year-olds felt most informed about general IBD knowledge, nutrition and drug treatment options but least informed about transition. The 14–15-year-old group felt most informed about IBD in general, nutrition and causes of IBD and they felt least informed about transition (Supplement S1).

Transition counseling usage and awareness vary by location: 52.1% of patients in small towns felt well-informed, compared to 38.4% in villages and just 9.6% in large cities. Parents in small towns were the most informed, with 58% reporting a good understanding of transition. In terms of regional differences, the western part of Germany had the highest proportion of both patients (37.8%) and parents (45.9%) who felt informed about transition. When considering information needs, the demand for more knowledge about transition was highest among patients living in small towns (46.3%) and villages (31.1%), while 22.6% of those in large cities expressed a desire for more information. Among parents, the need for additional knowledge was also highest in small towns (44.8%) and villages (29.6%). Regionally, the greatest need for information was reported by patients and parents in the west, where 35.2% of patients and only 2.1% of parents expressed a need for more information. The eastern and southern regions also reported a significant need for more information, with nearly 29% of patients and up to 40.3% of parents seeking further information (Table 1).

### 3.3. Treatment and Counseling Services Used by Patients and Parents of Patients

The survey examined the use of 15 different IBD treatment and counseling services by both patients and parents. Patients were asked which of these services they had personally received, while parents provided information on which services they had received for their child and which services they had received for themselves.

The results show that nearly half of the patients received nutritional counseling (49.2%, *n* = 382), about one-third of the patients reported not using any counseling services at all (31.4%, *n* = 379) and about one-third received psychological support (28.6%, *n* = 381). The least used services among patients were genetic counseling (2.1%, *n* = 381), self-help group services (2.1%, *n* = 381), transition programs (1.3%, *n* = 381), and sexual counseling (0.8%, *n* = 381).

For parents seeking services for their children, nutritional counseling (41.9%, *n* = 578) and psychological support (25.4%, *n* = 579) were the most commonly used services, mirroring the trends seen among patients. Similarly, the least used services for children were sexual counseling (0.0%, *n* = 579), transition programs (0.5%, *n* = 579), and self-help group services (2.6%, *n* = 579). In general, most parents did not use counseling services for their children (42.9%, *n* = 632).

**Figure 2:**
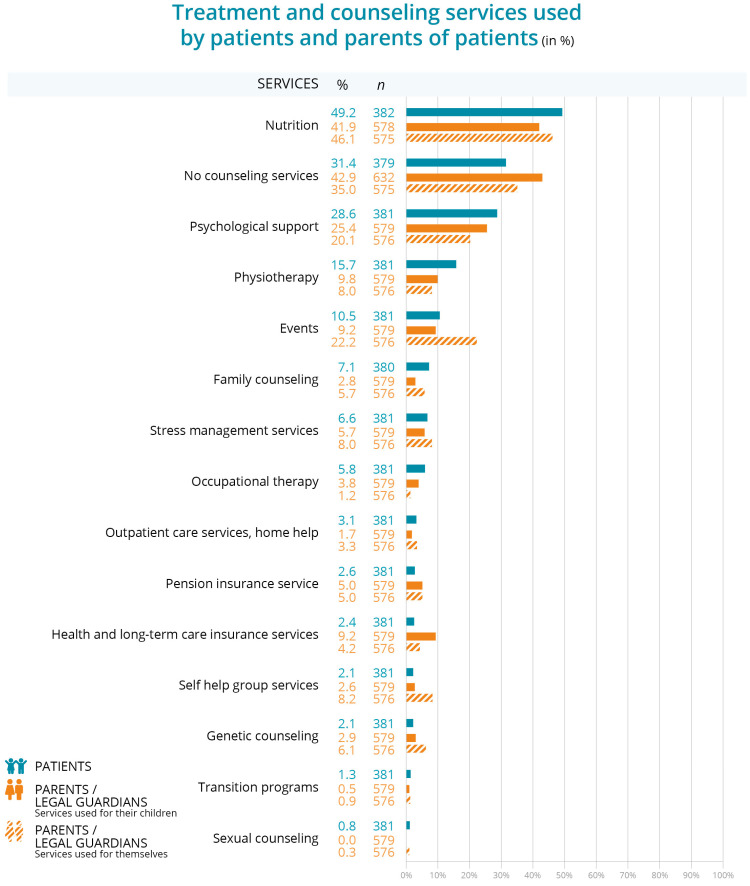
Utilization of counseling and treatment services for IBD by patients and parents of patients.

Among the services used by parents for themselves, the most common were nutrition counseling (46.1%, *n* = 575) and attendance at disease-related events (22.2%, *n* = 576). The least utilized services by parents for their children were sexual counseling (0.0%, n = 579) transition programs (0.5%, n = 579), and outpatient care services (1.7%, *n* = 579). More than one-third of parents (42.9%, *n* = 632) reported not using any counseling services for their own needs (Figure 2).

When the results were broken down into subgroups based on patient age (Supplement S2), it was found that nutrition counseling was received at similar rates in all age groups, with more than half of patients aged 12–13 years (54.5%, *n* = 287) and 14–15 years (56.8%, *n* = 287) receiving it, and slightly less in those aged 16–17 years (43.4%, *n* = 287). A similar pattern was observed among parents who received nutrition counseling for their children, with 43.8% (*n* = 419) of parents of 12–13-year-olds, 44.9% (*n* = 419) of parents of 14–15-year-olds, and 37.0% (*n* = 419) of parents of 16–17-year-olds receiving this service. Parents also accessed nutrition counseling for their own needs, with 51.0% (*n* = 414) of parents of 12–13-year-olds, 47.4% (*n* = 414) of 14–15-year-olds, and 35.8% (*n* = 414) of 16–17-year-olds reporting its use.

Psychological support was another important service, used by about a third of the patients themselves in all age groups (31.2%, *n* = 287 of 12–13-year-olds, 33.8%, *n* = 287 of 14–15-year-olds, and 33.1%, *n*= 287 of 16–17-year-olds). Parents also used this service for their children (31.4%, (*n* = 419) of 12–13-year-olds, 24.4%, (*n* = 419) of 14–15-year-olds, and 32.6%, (*n* = 419) of 16–17-year-olds). However, the use of psychological support by parents for their own needs was lower, with 26.9% (*n* = 415) of parents of 12–13-year-olds, 17.2% (*n* = 415) of 14–15-year-olds, and 16.8% (*n* = 415) of 16–17-year-olds making use of it.

Transition programs were not used at all in the age group of 12–13 age group (0.0%, *n* = 287) and only very little in the group of 14–15 age group (1.4%, *n* = 287) and in the oldest group of 16–17-year-olds (2.9%, *n* = 287).

The breakdown of use of transition counseling usage by region and place of residence reveals different patterns. We have five complete datasets on this topic. In terms of place of residence, 40% of patients living in small towns (*n* = 2) and large cities (*n* = 2) reported using transition counseling, while only 20% of those living in villages did so (*n* = 1). Among parents seeking transition counseling for their children (*n* = 5), two of those in large cities used these services, while one in small towns reported using them. Two parents in villages sought transition counseling for their children. Among parents seeking services for themselves (*n* = 3), one of those in villages and two in large cities used transition counseling, compared to zero in small towns.

Looking at regions, 60% of patients (*n* = 3) and 40% of parents (*n* = 2) living in the western states reported using transition counseling. In the eastern states, 20% of patients (*n* = 1) and 60% of parents (*n* = 3) used transition counseling for their children, and 33.3% of parents (*n* = 1) used the service for themselves. However, none of the patients or parents from northern Germany reported using transition counseling, and in the southern states, 20% of patients (*n* = 1) used these services, but no parents reported using them for themselves or their children.

**Figure 3:**
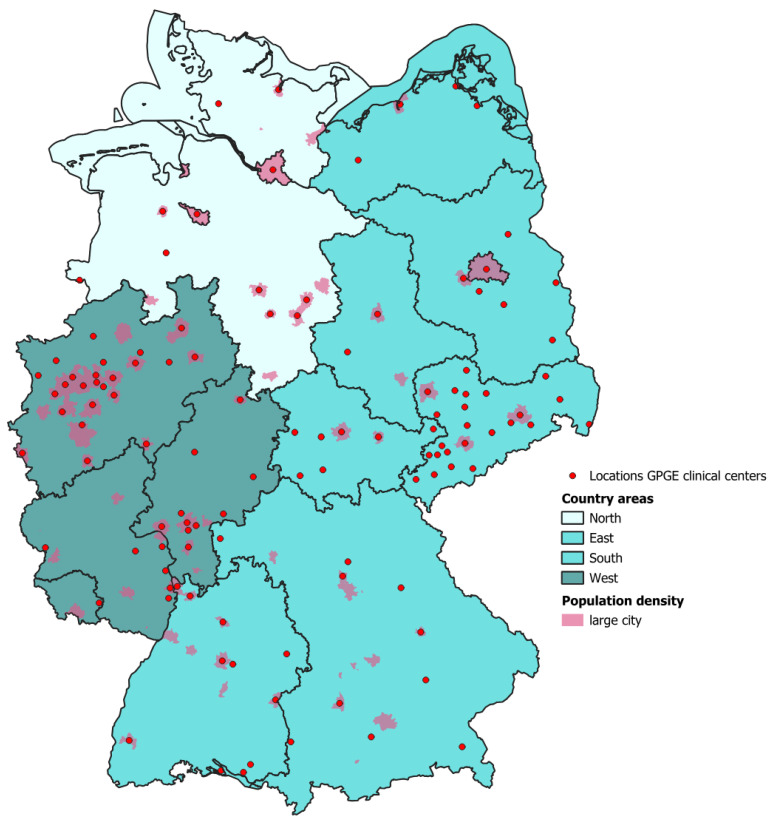
Use of treatment and counseling services by patients (aged 12–17) in transition to adult healthcare, broken down by region of residence in Germany.

This breakdown highlights regional disparities in the use of transition counseling services, with the western and eastern parts of the country showing higher engagement compared to the north and south. Additionally, place of residence plays a role, with patients and parents in larger cities more likely to access these services compared to those in smaller towns and villages (Figure 3).

### 3.4. Trustworthy Sources of Information About IBD

Patients and parents were asked whether they found the listed sources of information to be trustworthy. Doctors are overwhelmingly trusted, with 98.7% of patients (*n* = 310) and 95.1% of parents (*n* = 413) considering them a reliable source of information. This is closely followed by specialists’ journals and books, trusted by 79.5% of patients (*n* = 273) and 65.2% of parents (*n* = 342).

Nutritionists also ranked highly, being trusted by 74.5% of patients (*n* = 275) and 56.1% of parents (*n* = 335) viewing them as trustworthy. In addition, medical associations are trusted by 74.0% of patients (*n* = 268) but only 59.3% of parents (*n* = 330). Patients and parents have moderate levels of trust in other patients, 67.2% of patients (*n* = 271) and 46.9% of parents (*n* = 309). Patient associations and psychologists are similarly trusted, especially by patients (62.4%, *n* = 255 and 62.0%, *n* = 266).

**Figure 4:**
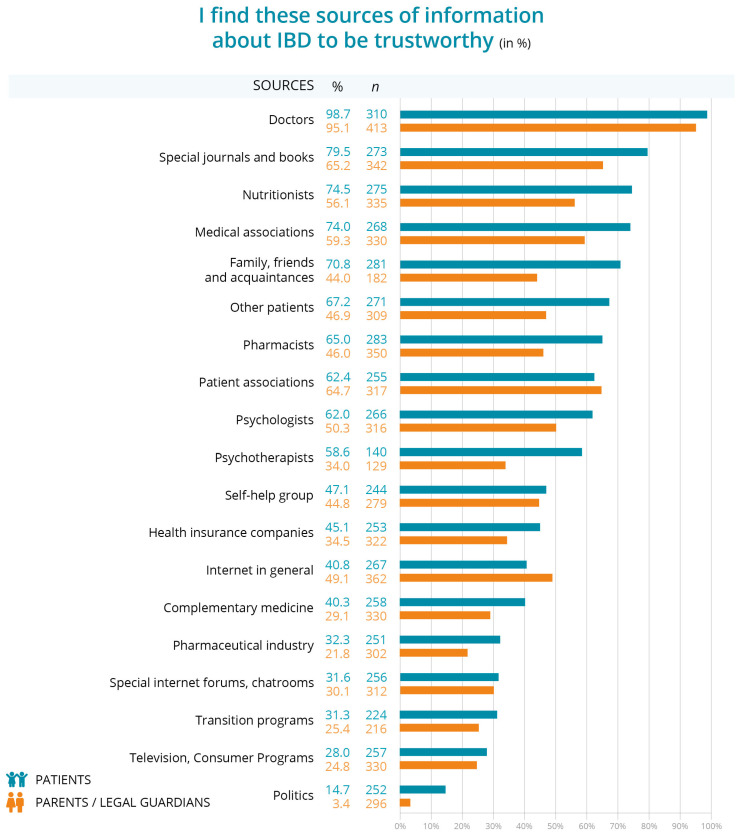
Trustworthiness of information sources on IBD.

Practitioners of complementary medicine are less trusted, with trust levels of 40.3% by patients (*n* = 258) and 29.1% by parents (*n* = 330). Self-help groups and health insurance companies are trusted by less than half of respondents. At the bottom of the list are politicians, television programs, and dedicated internet forums, which are trusted as reliable sources of IBD information by patients and parents. Transition programs come third to last with 31.3% of patients (*n* = 224) and 25.4% of parents (*n* = 216) finding them trustworthy (Figure 4). Practitioners of alternative medicine and the pharmaceutical industry are less trusted, with trust levels of around 20–40% for both patients and parents. Self-help groups and health insurance companies are trusted by less than half of respondents. At the bottom of the list are politicians, television programs, and dedicated internet forums, which are trusted as reliable sources of IBD information by 3.4% of parents and around 25–30% of patients. Transition programs come third to last with 31.3% of patients (*n* = 224) and 25.4% of parents (*n* = 216) finding them trustworthy (Figure 4).

The individual responses were broken down by patient age groups and time since diagnosis, with different levels of trust in different sources of information about IBD among patients aged 12–17 years and their parents, with clear trends based on age and disease duration (Supplement S3). Across all age groups and time since diagnosis, doctors are consistently considered the most trusted source by both patients and parents, with nearly 100% trust. Trust in other sources such as nutritionists, medical associations, and family friends varies significantly between patients and parents, and generally declines with age. Parents tend to be more skeptical of sources such as pharmacists, psychologists, and practitioners of alternative medicine than patients, while both groups show low levels of trust in information from pharmaceutical companies, the internet, and political sources. Trust in information provided by self-help groups and special internet forums also declines over time, especially among parents.

**Figure 5:**
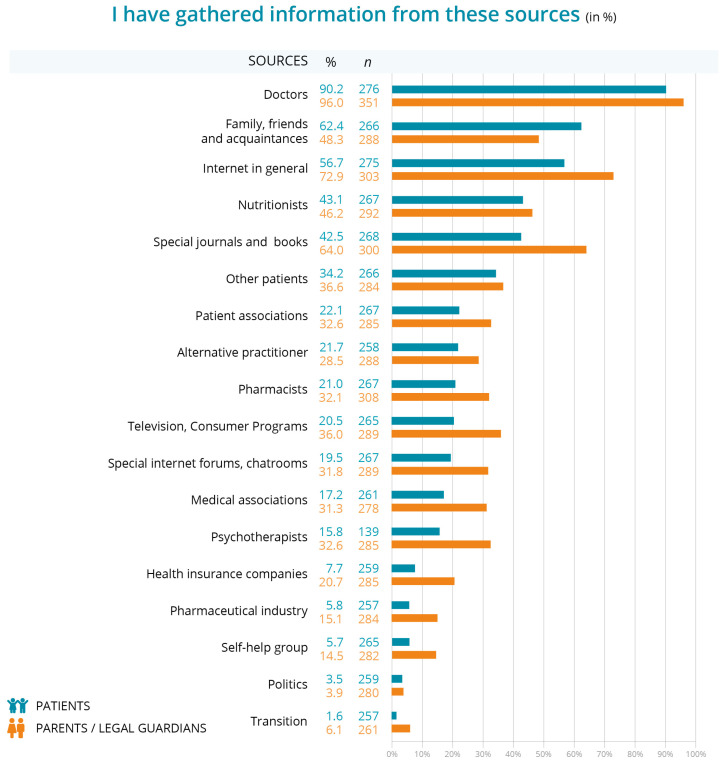
Information sources used by patients and parents of patients.

Patients and parents were also asked to indicate whether they had already sought information from the sources listed (Figure 5). Doctors are the most commonly used source, with 96.2% of respondents saying that they consulted them. Family, friends and acquaintances and the internet in general are also frequently used, with usage rates of 62.4% and 56.7%, respectively. Nutritionists and specialist journals/books are consulted by about 43.1% and 42.5% of the respondents, showing moderate engagement. Notably, there is significant use of other patients (34.2%) and patient associations (22.1%). Less than 6% of respondents consulted sources such as pharmaceutical industry information, self-help groups, political channels, or transition programs, indicating a low reliance on these types of information.

Regarding the trustworthiness of the information provided by transition programs, a subgroup analysis was conducted according to place of residence and region. Trust in transition programs is generally low, with the highest trust expressed by patients from small towns (38.5%, *n* = 27) and the western regions (37.1%, *n* = 26). Parents show similar trust (small towns 43.7%, *n* = 24 and western regions (43.6%, *n* = 24). Use of information from transition programs is extremely limited (patients *n* = 4, parents *n* = 15), with most regions and locations showing near zero engagement. Patient engagement is highest in small towns and big cities (50%) and the eastern region (50%), while parents also show minimal usage, with slightly higher rates in the western regions (40%). Overall, both trust and use of transition program information are very low across all demographics.

### 3.5. Timing of Information on IBD

A presentation of all relevant topics related to IBD and the assessment of the appropriate timing of information requested by parents has been shown in a previous publication [20]. Parents’ perspectives on when they need information about IBD for their children, segmented by place and region of residence, were analyzed (Table 2). Most parents from small towns expressed a need for information at diagnosis (47.4%) and in the first year of diagnosis (52.2%). In contrast, parents from large cities have the lowest need for information in the first year (8.7%). Regionally, parents in the southern and western regions have a higher need for information at diagnosis (42.1% and 15.8%, respectively), while those in the west have the highest need in the first year (47.8%).

Regarding the need for information during the ongoing course of the disease, most parents from small towns (47.3%) need ongoing information, while parents from large cities have the lowest need (22.9%). Regionally, parents in the west (34.9%) and south (30.7%) are more likely to need ongoing information. Patients need the most information at the time of diagnosis and throughout the first year, especially parents from small towns in southern and western Germany.

## 4. Discussion

The primary aim of the CEDNA survey was to determine the current care landscape for children and adolescents with PIBD in Germany. The state of research regarding the additional support needs of affected patients and their families has so far been inadequate. To address this deficit, a comprehensive analysis of the survey results regarding the disease, medical and psychological care, coping with the disease, the level of knowledge about PIBD and the search for information was published for the first time [20,21]. These data serve as a basis for the creation of a database that will facilitate the communication process between healthcare teams, patients and other stakeholders with the aim of strengthening patient empowerment [20]. One of the key findings from the preliminary analysis of the CEDNA survey was the limited use of counseling services by patients and their parents. As a second step, the data were analyzed in more detail and the results presented here.

Patients and parents feel most competent about general IBD information, nutrition, and available drug treatments. Patients stated that they were well informed about nutrition in particular, and nutritional advice was also the most frequently requested support service. One reason for this could be that communication about nutrition is omnipresent in the media [22].

However, the transition process is an area where knowledge is significantly lacking, with only 25.2% of patients and 9.1% of parents feeling informed. Patients aged 16–17 and those diagnosed for 1–2 years increasingly seek more information about the transition to adult care as they get older. Parents and patients from different locations have different levels of knowledge and information needs about transition. Patients in small towns report the highest level of knowledge (52.1%), while those in large cities report the lowest (9.6%). Similarly, parents in small towns feel more informed (58%) compared to those in villages or large cities. Regionally, western Germany has the highest level of knowledge about transition between both patients (37.8%) and parents (45.9%). In small towns, the demand for information about the handover process is highest, with 46.3% of patients and 44.8% of parents wanting more details. Regionally, the need is greatest in the western and southern parts of Germany, where more than 35.2% of patients and up to 40.3% of parents want more information.

Overall, while knowledge about general IBD topics is relatively high, there is a considerable gap in understanding and a strong need for information about the transition process, particularly in certain demographic groups. These findings suggest a clear gap in the current provision of transition-related information. Patients seem well-informed about managing their condition medically, but there is a clear gap in preparing them for the practical und emotional transition from pediatric to adult healthcare. This gap is problematic, as poor preparation for transition can lead to anxiety, discontinuity of care, and suboptimal disease management once patients enter the adult healthcare system [19]. The data also point to the need for healthcare providers to engage patients and their families in transition discussions at an earlier stage. Transition is a complex process that requires not only medical knowledge but also psychological and emotional preparation [23]. Adolescents and their families must be equipped with the necessary tools and resources to successfully manage this shift, and healthcare providers should prioritize educating them on the logistical, medical, and emotional aspects of transitioning to adult care [5,23]. Analysis of the data revealed geographic differences in patient transition. Structured transitions are more likely to occur in large cities. A possible reason for this could be the availability of larger clinics and specialized professionals in urban areas who can provide more comprehensive care and support. These circumstances underscore the need to improve the accessibility and visibility of counseling services in rural areas in order to ensure adequate support for all affected families.

Furthermore, this lack of specialist staff may also be a reason why many patients stated that they did not make use of counseling services at all [24]. The feelings of children and adolescents can also lead to them not wanting to share their concerns and therefore generally not making use of counseling services [24]. Low transition program usage rates from pediatric to adult healthcare are not unique to Germany. It is a global issue, observed across a variety of healthcare systems and countries, including the USA [25] and the United Kingdom [26]. A 2020 report by the National Survey of Children’s Health found that only about 15–20% of Routh with special healthcare needs received transition planning as recommended [25]. The NHS Long-Term Plan recognized transition care as a weak point [26]. Many youths experience gaps or even complete loss of care after leaving pediatric services.

The results on the availability of counseling services show that parents are more likely than children and adolescents to use information sessions to learn about the disease and how to manage it. Nearly half of patients used nutritional counseling (49.2%), about one-third reported no use of counseling services (31.4%), and a similar proportion used psychological support (28.6%); the least used services were genetic counseling (2.1%) and self-help group services (2.1%). Parents also frequently sought nutritional counseling (41.9%) and psychological support (25.4%) for their children, while 42.9% did not seek counseling services. Nutritional counseling (46.1%) and attendance at disease-related events (22.2%) were also commonly used services among parents. Across the different age groups of patients, especially 12–17 years, the use of nutritional counseling remained consistently high. A third of patients utilized psychological support, whereas parents were less likely to seek it for themselves. Use of transition programs varied significantly by region, with urban areas showing higher use than rural areas.

Counseling services for children and adolescents with PIBD and affected parents are of central importance for the support these groups of people [27]. Nutritional advice and psychological support are particularly important, as they correlate directly with the quality of life of affected patients [28,29]. These services are usually available in hospitals, which can make them easier to access and use [30]. Close collaboration with healthcare professionals enables families to make informed decisions and better manage the challenges associated with IBD. One possible explanation for the low level of interest may be the initiative that is often required to take time off work to participate in such programs. This may mean that many patients and their families do not see the benefits of these services or do not consider them useful.

Our results show that trust in the counseling services provided by self-help groups and transition programs is low, compared to other forms of counseling. Seventy percent of parents and 59.3% of patients trust family, friends and acquaintances. The group of children and adolescents had even less trust in self-help groups (34.0%), which were rated as trustworthy by 47.1% of the parents concerned. Both children and adolescents (30.1%) and parents (31.1%) rated transition programs as less trustworthy. In particular, the analysis of the data, evaluated according to the age of the children and adolescents concerned and the duration of the diagnosis, shows that trust in transition programs does not increase over the years. After more than 6 years of diagnosis, it is 34% for children and adolescents and only 12.1% for parents. There may be several reasons for these findings. A key issue may be that the expectations of affected patients may be different from those offered by existing programs [31]. Children and adolescents have specific needs and expectations that may not be adequately addressed in the current programs [31]. This finding suggests that a review and adaptation of existing programs may be necessary. A more targeted and needs-oriented approach could not only better meet the needs of children and adolescents, but also strengthen their loyalty to support programs. Future research should therefore examine in more detail which specific elements of program development are appropriate for the target group to enable sustainable trust in the counseling services.

The results of the survey show that it makes a difference which information about IBD is requested at which point in the disease. Whether the place and region of residence also have an influence in this regard was also evaluated. Parents from small towns most frequently require information about the diagnosis (47.4%) and in the first year (52.2%). In contrast, parents from large cities have the lowest need for information in the first year (8.7%). Regional differences show that parents in the south and west have a greater need for information about the diagnosis. Overall, the need for information is particularly pronounced at the beginning of the disease, especially among parents in small towns and in the south and the west of Germany. This result corresponds to the current state of research on the topic of health literacy [32]. People with chronic illnesses have a great interest in health information [33]. A better understanding of the disease of PIBD, its treatment options and the support services available is essential in order to enable patients to adopt an appropriate self-management strategy and improve their quality of life in the long term [34].

Limitations of the study are described in [20]. The degree of missing or incorrect responses varied across the surveys. For the patient questionnaires, the median number of missing or incorrect answers was 0.15, with an interquartile range of 0.09 to 0.19. For the parent questionnaires, the median was slightly higher at 0.22, with an interquartile range of 0.08 to 0.27.

## 5. Conclusions

There is a significant gap in knowledge and preparedness for transition among IBD patients, particularly in the 12–17 age group. While general knowledge about managing IBD is relatively good, targeted efforts are needed to improve awareness and preparedness for the transition process. The results show that potential sources of information need to be trustworthy and that information on disease-related topics is sought at different points in the disease course. Early education and support programs tailored to these different stages could contribute to a smoother transition and ultimately lead to better long-term disease management and improved patient outcomes in adult care. Closer cooperation between pediatric and adult care for patients with IBD therefore seems sensible and necessary. The fixed age of 18 years also appears to be too restrictive in this context.

To address the specific needs of the patient group with PIBD, establishing a nationwide, standardized information service appears to be urgently necessary. The service must rely on the latest scientific research and be updated regularly to keep patients fully informed. A standardized information portal could ensure that all patients, regardless of where they live, have access to high quality information about IBD. It could include interactive elements such as question-and-answer sessions, webinars with experts and platforms for patient dialog. The implementation of such resources could help to improve understanding of the disease and promote dialog among those affected. Specific interventions can be developed for this, such as transition workshops and peer mentoring. In addition, the benefits of rural outreach programs should be further explored, as urban areas seem more accessible. The use of, for example, testimonials from previous participants as confidence-building measures can also be explored in further research.

## Figures and Tables

**Table 1:** Level of knowledge and information needs of patients (aged 12–17 years) and parents of patients (aged 0–17 years) on transition, broken down by place of residence (village: <5000 inhabitants; small town: 5000–100,000 inhabitants; large city: >100,000) and region of residence in Germany.

Responses in %		
	Patients	Parents
**Level of knowledge (I feel informed about the transition)**		
**Place of residence**	*n = 73*	*n = 88*
Village	38.4	28.4
Small town	52.1	58.0
Large city	9.6	13.6
**Region of residence**	*n = 74*	*n = 89*
North	12.2	9.0
East	23.0	16.9
South	27.0	29.2
West	37.8	45.9
**Information needs (I would like to know more about transition)**		
**Place of residence**	*n = 164*	*n = 67*
Village	31.1	29.6
Small town	46.3	44.8
Large city	22.6	28.4
**Region of residence**	*n = 165*	*n = 67*
North	6.7	6.0
East	29.7	32.8
South	28.5	40.3
West	35.2	2.1

**Table 2:** Timing of information on IBD as assessed by parents of patients on transition, broken down by place of residence and region of residence.

Responses in %			
	Parents		Parents
**I need information at diagnosis**		**I need information in the first year of diagnosis**	
**Place of residence**	*n = 19*	**Place of residence**	*n = 23*
Village	21.0	Village	39.1
Small town	47.4	Small town	52.2
Big city	31.6	Big city	8.7
**Region of residence**	*n = 19*	**Region of residence**	*n = 23*
North	5.3	North	4.3
East	36.8	East	17.4
South	42.1	South	30.4
West	15.8	West	47.8
**I need information during the further disease course**		**I have no need for information**	
**Place of residence**	*n = 433*	**Place of residence**	*n = 41*
Village	29.8	Village	22.0
Small town	47.3	Small town	58.5
Big city	22.9	Big city	19.5
**Region of residence**	*n = 433*	**Region of residence**	*n = 41*
North	11.5	North	9.8
East	22.9	East	7.3
South	30.7	South	29.3
West	34.9	West	53.7

## Data Availability

The datasets were obtained from the quality improvement project CED-KQN (Big Data eHealth—Improving the Care of Children and Adolescents with IBD) which was funded by the Federal Joint Innovation Committee G-BA and is made available on the G-BA’s URL https://innovationsfonds.g-ba.de/projekte/versorgungsforschung/ced-kqn-big-data-ehealth-verbesserung-der-versorgung-von-kindern-und-jugendlichen-mit-chronisch-entzuendlichen-darmerkrankungen.171 (accessed on 20 February 2025).

## Supplementary Materials

The following supporting information can be downloaded at: https://www.mdpi.com/article/10.3390/children12050620/s1, Supplement S1: Topics about which patients (aged 12–17 years) and parents of patients feel or don’t feel well informed. Supplement S2: Counseling services used by IBD patients (aged 12–17 years) and by parents of IBD patients for their children or for themselves, broken down by age of the patients (12–17 years). Supplement S3: Trustworthiness of information sources on IBD as assessed by patients (aged 12–17 years) and parents of patient, broken down by age and duration since diagnosis.
