# Supplementary material for: Patient Empowerment Among Children and Adolescents with Inflammatory Bowel Disease (IBD) and Parents of IBD Patients—Use of Counseling Services and Lack of Knowledge About Transition
Source: Children (Basel). 2025 May 10;12(5):620. doi: 10.3390/children12050620 (PMC12110410; doi:10.3390/children12050620)
Supplement: Supplementary file 1 [file children-12-00620-s001.zip › Supplement S1.pdf]

Supplement S1: Topics about which patients (aged 12-17 years) and parents of patients feel or don't feel well informed. Presentation of all relevant topics related to IBD and survey respondents' ratings of whether or not they feel well informed about these topics, broken down by age and time since diagnosis.

Responses (Yes, I feel well informed on this topic; No, I don't feel well informed on this topic) in %

Topics	Patients		Parents			Patients		Parents	
**IBD in general**	**Yes**	**No**	**Yes**	**No**	**Nutrition**	**Yes**	**No**	**Yes**	**No**
Age	*n=240*		*n=409*		Age	*n=239*		*n=387*	
12-13 years	25.3	74.7	16.6	83.4	12-13 years	21.9	78.1	14.5	85.5
14-15 years	25.3	74.7	28.1	71.9	14-15 years	23.7	76.3	24.6	75.4
16-17 years	36.2	63.8	21.8	78.2	16-17 years	32.6	67.4	18.3	81.7
Disease duration	*n=308*		*n=410*		Disease duration	*n=297*		*n=288*	
<1 year	11.4	88.6	15.9	84.1	<1 year	9.4	90.6	7.2	92.8
1-2 years	25.0	75.0	23.7	76.3	1-2 years	22.9	77.1	13.9	86.1
3-4 years	19.8	80.2	21.0	79.0	3-4 years	18.9	81.1	13.7	86.3
5-6 years	12.7	87.3	10.2	89.8	5-6 years	11.8	88.2	6.2	93.8
>6 years	17.9	82.1	17.6	82.4	>6 years	17.5	82.5	8.8	91.2
**Drug treatment options**	**Yes**	**No**	**Yes**	**No**	**Vaccinations**	**Yes**	**No**	**Yes**	**No**
Age	*n=240*		*n=386*		Age	*n=241*		*n=396*	
12-13 years	21.9	78.1	14.8	85.2	12-13 years	17.7	82.3	14.1	85.9
14-15 years	19.2	80.8	24.6	75.4	14-15 years	20.4	79.6	24.5	75.5
16-17 years	32.0	68.0	19.2	80.8	16-17 years	29.6	70.4	15.7	84.3
Disease duration	*n=298*		*n=387*		Disease duration	*n=301*		*n=396*	
<1 year	8.7	91.3	13.7	86.3	<1 year	8.3	91.7	12.1	87.9
1-2 years	18.3	81.7	19.3	80.7	1-2 years	17.6	82.4	16.9	83.1
3-4 years	14.0	86.0	19.6	80.4	3-4 years	15.3	84.7	17.7	82.3
5-6 years	9.7	90.3	10.3	89.7	5-6 years	11.0	89.0	8.1	91.9
>6 years	14.3	85.7	14.2	85.8	>6 years	16.0	84.0	14.4	85.6
**Side effects of medication**	**Yes**	**No**	**Yes**	**No**	**School and education**	**Yes**	**No**	**Yes**	**No**
Age	*n=240*		*n=389*		Age	*n=238*		*n=375*	
12-13 years	17.0		12.1	87.9	12-13 years	17.7	82.3	8.0	92.0
14-15 years	17.9		21.6	78.4	14-15 years	17.7	82.3	18.7	81.3
16-17 years	28.6		13.9	86.1	16-17 years	27.0	73.0	12.8	87.2
Disease duration	*n=300*		*n=387*		Disease duration	*n=300*		*n=387*	
<1 year	9.0		13.7	86.3	<1 year	9.3	90.7	8.2	91.8
1-2 years	21.8		19.4	80.6	1-2 years	14.0	86.0	10.6	89.4
3-4 years	20.5		19.6	80.4	3-4 years	14.3	85.7	13.2	86.8
5-6 years	10.4		10.3	89.7	5-6 years	10.2	89.8	4.8	95.2
>6 years	9.1		14.2	85.8	>6 years	16.0	84.0	11.1	88.9
**Prognosis**	**Yes**	**No**	**Yes**	**No**	**Sexuality**	**Yes**	**No**	**Yes**	**No**
Age	*n=238*		*n=379*		Age	*n=238*		*n=378*	
12-13 years	15.9	84.1	12.1	87.9	12-13 years	11.7	88.3	7.1	92.9
14-15 years	18.2	81.8	19.5	80.5	14-15 years	14.4	85.6	11.6	88.4
16-17 years	27.7	72.3	13.5	86.5	16-17 years	24.8	75.2	9.3	90.7
Disease duration	*n=296*		*n=379*		Disease duration	*n=295*		*n=378*	
<1 year	8.5	91.5	9.5	90.5	<1 year	6.1	93.9	4.8	95.2
1-2 years	18.2	81.8	16.9	83.1	1-2 years	13.6	86.4	8.5	91.5
3-4 years	13.5	86.5	13.7	86.3	3-4 years	11.5	88.5	9.0	91.0
5-6 years	9.8	90.2	7.9	92.1	5-6 years	6.8	93.2	4.8	95.2
>6 years	11.5	88.5	11.4	88.6	>6 years	14.6	85.4	6.9	93.1

Dealing with psychological stress	Yes	No	Yes	No	Causes for IBD	Yes	No	Yes	No
Age	*n*=239		*n*=381		Age	*n*=241		*n*=373	
12-13 years	14.3	85.7	10.0	90.0	12-13 years	14.9	85.1	11.5	88.5
14-15 years	14.3	85.7	17.8	82.2	14-15 years	22.8	77.2	21.7	78.3
16-17 years	24.6	75.4	12.1	87.9	16-17 years	19.1	80.9	14.5	85.5
Disease duration	*n*=294		*n*=381		Disease duration	*n*=298		*n*=374	
<1 year	7.1	92.9	8.1	91.9	<1 year	6.0	94.0	11.0	89.0
1-2 years	14.0	86.0	11.8	88.2	1-2 years	14.1	85.9	14.4	85.6
3-4 years	11.6	88.4	14.4	85.6	3-4 years	10.4	89.6	15.5	84.5
5-6 years	7.8	92.2	5.5	94.5	5-6 years	9.1	90.9	8.8	91.2
>6 years	7.1	92.9	10.5	89.5	>6 years	9.4	90.6	14.2	85.8
**Psychotherapeutic support measures**	**Yes**	**No**	**Yes**	**No**	**Complications in the disease progress**	**Yes**	**No**	**Yes**	**No**
Age	*n*=236		*n*=386		Age	*n*=241		*n*=385	
12-13 years	13.4	86.6	9.3	90.7	12-13 years	15.0	85.0	10.4	89.6
14-15 years	14.3	85.7	13.2	86.8	14-15 years	11.0	89.0	17.4	82.6
16-17 years	22.1	77.9	10.1	89.9	16-17 years	21.1	78.9	11.4	88.6
Disease duration	*n*=288		*n*=387		Disease duration	*n*=304		*n*=386	
<1 year	5.6	94.4	5.9	94.1	<1 year	4.9	95.1	9.1	90.9
1-2 years	11.5	88.5	11.6	88.4	1-2 years	11.2	88.8	14.3	85.7
3-4 years	11.5	88.5	12.1	87.9	3-4 years	11.8	88.2	10.9	89.1
5-6 years	6.3	93.7	4.4	95.6	5-6 years	8.6	91.4	5.4	94.6
>6 years	5.6	94.4	9.6	90.4	>6 years	4.9	95.1	11.7	88.3
**Preventive health care**	**Yes**	**No**	**Yes**	**No**	**Concomitant diseases**	**Yes**	**No**	**Yes**	**No**
Age	*n*=241		*n*=373		Age	*n*=239		*n*=383	
12-13 years	10.4	89.6	11.0	89.0	12-13 years	12.5	87.5	9.6	90.4
14-15 years	14.4	85.6	16.9	83.1	14-15 years	10.7	89.3	18.3	81.7
16-17 years	19.8	80.2	11.3	88.7	16-17 years	18.3	81.7	11.2	88.8
Disease duration	*n*=294		*n*=374		Disease duration	*n*=300		*n*=384	
<1 year	4.1	95.9	8.8	91.2	<1 year	5.3	94.7	7.6	92.4
1-2 years	10.5	89.5	13.9	86.1	1-2 years	8.6	91.4	13.3	86.7
3-4 years	11.2	88.8	11.2	88.8	3-4 years	8.9	91.1	11.5	88.5
5-6 years	8.2	91.8	7.0	93.0	5-6 years	6.6	93.4	5.2	94.8
>6 years	10.9	89.1	9.6	90.4	>6 years	5.3	94.7	12.0	88.0
**Surgical treatment options**	**Yes**	**No**	**Yes**	**No**	**Patient organizations and self-help groups**	**Yes**	**No**	**Yes**	**No**
Age	*n*=237		*n*=394		Age	*n*=238		*n*=382	
12-13 years	10.4	89.6	7.9	92.1	12-13 years	9.4	90.6	7.6	92.4
14-15 years	8.1	91.9	15.2	84.8	14-15 years	12.1	87.9	15.7	84.3
16-17 years	22.6	77.4	9.4	90.6	16-17 years	17.9	82.1	9.9	90.1
Disease duration	*n*=299		*n*=395		Disease duration	*n*=272		*n*=393	
<1 year	2.7	97.3	6.1	93.9	<1 year	5.2	94.8	6.6	93.4
1-2 years	9.4	90.6	9.9	90.1	1-2 years	7.4	92.6	10.2	89.8
3-4 years	11.0	89.0	10.9	89.1	3-4 years	7.7	92.3	9.2	90.8
5-6 years	6.7	93.3	4.3	95.7	5-6 years	4.0	96.0	5.3	94.7
>6 years	2.7	97.3	6.1	93.9	>6 years	5.2	94.8	8.1	91.9
**Travel abroad**	**Yes**	**No**	**Yes**	**No**	**Complementary medicine**	**Yes**	**No**	**Yes**	**No**
Age	*n*=240		*n*=377		Age	*n*=240		*n*=367	
12-13 years	8.0	92.0	5.6	94.4	12-13 years	6.4	93.6	7.1	92.9
14-15 years	9.4	90.6	11.7	88.3	14-15 years	8.6	91.4	10.1	89.9

16-17 years	16.5	83.5	9.3	90.7	16-17 years	14.1	85.9	6.0	94.0
Disease duration	*n=295*		*n=377*		Disease duration	*n=292*		*n=368*	
<1 year	4.8	95.2	4.0	96.0	<1 year	2.7	97.3	5.2	94.8
1-2 years	7.1	92.9	7.7	92.3	1-2 years	6.9	93.1	6.8	93.2
3-4 years	6.4	93.6	10.1	89.9	3-4 years	7.5	92.5	7.6	92.4
5-6 years	5.8	94.2	5.0	95.0	5-6 years	4.5	95.5	3.5	96.5
>6 years	10.5	89.5	7.4	92.6	>6 years	2.7	97.3	7.6	92.4
**Social legal issues**	**Yes**	**No**	**Yes**	**No**	**Transition**	**Yes**	**No**	**Yes**	**No**
Age	*n=236*		*n=363*		Age	*n=239*		*n=360*	
12-13 years	10.1	89.9	4.4	95.6	12-13 years	4.1	95.9	5.6	94.4
14-15 years	6.9	93.1	10.7	89.3	14-15 years	6.8	93.2	7.5	92.5
16-17 years	11.9	88.1	7.2	92.8	16-17 years	14.1	85.9	6.7	93.3
Disease duration	*n=286*		*n=364*		Disease duration	*n=292*		*n=361*	
<1 year	4.6	95.4	4.1	95.9	<1 year	2.1	97.9	3.6	96.4
1-2 years	6.3	93.7	6.9	93.1	1-2 years	6.9	93.1	5.3	94.7
3-4 years	9.1	90.9	7.1	92.9	3-4 years	5.8	94.2	7.5	92.5
5-6 years	3.9	96.1	3.3	96.7	5-6 years	3.8	96.2	3.3	96.7
>6 years	4.6	95.4	7.7	92.3	>6 years	6.2	93.8	5.3	94.7
