# Supplementary material for: Patient Empowerment Among Children and Adolescents with Inflammatory Bowel Disease (IBD) and Parents of IBD Patients—Use of Counseling Services and Lack of Knowledge About Transition
Source: Children (Basel). 2025 May 10;12(5):620. doi: 10.3390/children12050620 (PMC12110410; doi:10.3390/children12050620)
Supplement: Supplementary file 1 [file children-12-00620-s001.zip › Supplement S2.pdf]

Supplement S2: Counseling services used by IBD patients (aged 12-17 years) and by parents of IBD patients for their children or for themselves, broken down by age of the patients (12-17 years)

Responses (Yes, I used these counseling services / No, I didn't use these counseling services) in %													
**Topics**	**Patients**		**Parents for their children**		**Parents for themselves**			**Patients**		**Parents for their children**		**Parents for themselves**	
**Nutrition**	**Yes**	**No**	**Yes**	**No**	**Yes**	**No**	**No counseling services**	**Yes**	**No**	**Yes**	**No**	**Yes**	**No**
Age	*n=287*		*n=419*		*n=414*		Age	*n=286*		*n=418*		*n=414*	
12-13 years	54.5	45.5	43.8	56.2	51.0	49.0	12-13 years	27.3	72.7	39.0	61.0	33.7	66.3
14-15 years	56.8	43.2	44.9	55.1	47.4	52.6	14-15 years	31.5	68.5	36.9	63.1	34.7	65.3
16-17 years	43.4	56.6	37.0	63.0	35.8	64.2	16-17 years	32.4	67.6	32.8	67.2	37.2	62.8
**Psychological support**	**Yes**	**No**	**Yes**	**No**	**Yes**	**No**	**Physiotherapy**	**Yes**	**No**	**Yes**	**No**	**Yes**	**No**
Age	*n=287*		*n=419*		*n=415*		Age	*n=287*		*n=419*		*n=415*	
12-13 years	31.2	68.8	31.4	68.6	26.9	73.1	12-13 years	10.4	89.6	8.6	91.4	3.8	96.2
14-15 years	33.8	66.2	24.4	75.6	17.2	82.8	14-15 years	20.3	79.7	9.1	90.9	9.8	90.2
16-17 years	33.1	66.9	32.6	67.4	16.8	83.2	16-17 years	16.9	83.1	12.3	87.7	8.0	92.0
**Events**	**Yes**	**No**	**Yes**	**No**	**Yes**	**No**	**Family counseling**	**Yes**	**No**	**Yes**	**No**	**Yes**	**No**
Age	*n=287*		*n=419*		*n=415*		Age	*n=286*		*n=419*		*n=415*	
12-13 years	3.9	96.1	6.7	93.9	17.3	82.7	12-13 years	9.1	90.9	1.9	98.1	4.8	95.2
14-15 years	10.8	89.2	9.7	90.3	27.0	73.0	14-15 years	9.5	90.5	1.1	98.9	3.4	96.6
16-17 years	12.5	87.5	16.7	83.3	26.3	73.7	16-17 years	5.9	94.1	6.5	93.5	8.8	91.2
**Stress management services**	**Yes**	**No**	**Yes**	**No**	**Yes**	**No**	**Occupational therapy**	**Yes**	**No**	**Yes**	**No**	**Yes**	**No**
Age	*n=287*		*n=419*		*n=415*		Age	*n=287*		*n=419*		*n=415*	
12-13 years	5.2	94.8	4.8	95.2	7.7	92.3	12-13 years	3.9	96.1	3.8	96.2	0.0	100.0
14-15 years	8.1	91.9	4.0	96.0	7.5	92.5	14-15 years	9.5	90.5	4.0	96.0	1.7	98.3
16-17 years	6.6	93.4	10.1	89.9	10.2	10.2	16-17 years	4.4	95.6	2.2	97.8	0.7	99.3
**Outpatient care services, home help**	**Yes**	**No**	**Yes**	**No**	**Yes**	**No**	**Pension insurance services**	**Yes**	**No**	**Yes**	**No**	**Yes**	**No**
Age	*n=287*		*n=419*		*n=415*		Age	*n=287*		*n=419*		*n=415*	
12-13 years	2.6	97.4	1.0	99.0	4.8	95.2	12-13 years	5.2	94.8	8.6	91.4	6.7	93.3
14-15 years	2.7	97.3	2.3	97.7	3.4	96.6	14-15 years	2.7	97.3	2.8	97.2	5.2	94.8
16-17 years	3.7	96.3	1.4	98.6	2.2	97.8	16-17 years	2.9	97.1	4.3	95.7	2.9	97.1
**Health and long-term care insurance**	**Yes**	**No**	**Yes**	**No**	**Yes**	**No**	**Self-help group services**	**Yes**	**No**	**Yes**	**No**	**Yes**	**No**
Age	*n=287*		*n=419*		*n=415*		Age	*n=287*		*n=419*		*n=415*	
12-13 years	3.9	96.1	1.9	98.1	4.8	95.2	12-13 years	1.3	98.7	2.9	97.1	11.5	88.5
14-15 years	1.4	98.6	1.1	98.9	5.2	94.8	14-15 years	1.4	98.6	2.3	97.7	6.9	93.1
16-17 years	2.2	97.8	1.4	98.6	3.6	96.4	16-17 years	2.9	97.1	4.3	95.7	7.3	92.7
**Genetic counseling**	**Yes**	**No**	**Yes**	**No**	**Yes**	**No**	**Transition programs**	**Yes**	**No**	**Yes**	**No**	**Yes**	**No**
Age	*n=287*		*n=419*		*n=415*		Age	*n=287*		*n=419*		*n=415*	
12-13 years	1.3	98.7	1.0	99.0	3.8	96.2	12-13 years	0.0	100.0	0.0	100.0	0.0	100.0
14-15 years	2.7	97.3	1.1	98.9	3.4	96.6	14-15 years	1.4	98.6	0.0	100.0	1.1	98.9
16-17 years	2.2	97.8	0.0	100.0	2.2	97.8	16-17 years	2.9	97.1	2.2	97.8	2.2	97.8

Sex Counseling	Yes	No	Yes	No	Yes	No	Family planning counseling	Yes	No	Yes	No	Yes	No
Age	*n=287*		*n=419*		*n=415*		Age			*n=419*		*n=415*	
12-13 years	1.3	98.7	0.0	100.0	0.0	100.0	12-13 years	-	-	0.0	100.0	0.0	100.0
14-15 years	0.0	100.0	0.0	100.0	1.1	98.9	14-15 years	-	-	0.0	100.0	1.1	98.9
16-17 years	0.7	99.3	0.0	100.0	0.0	100.0	16-17 years	-	-	0.0	100.0	0.7	99.3
