# Supplementary material for: Patient Empowerment Among Children and Adolescents with Inflammatory Bowel Disease (IBD) and Parents of IBD Patients—Use of Counseling Services and Lack of Knowledge About Transition
Source: Children (Basel). 2025 May 10;12(5):620. doi: 10.3390/children12050620 (PMC12110410; doi:10.3390/children12050620)
Supplement: Supplementary file 1 [file children-12-00620-s001.zip › Supplement S3.pdf]

Supplement S3: Trustworthiness of information sources on IBD as assessed by patients (aged 12-17 years) and parents of patient, broken down by age and duration since diagnosis.

Responses (I think these sources are trustworthy) in %

Topics	Patients		Parents		Topics	Patients		Parents	
**Doctors**	**Yes**	**No**	**Yes**	**No**	**Special journals and books**	**Yes**	**No**	**Yes**	**No**
Age	*n=233*		*n=289*		Age	*n=209*		*n=249*	
12-13 years	28.3	0.0	26.5	0.3	12-13 years	17.2	8.6	18.5	9.2
14-15 years	27.0	0.4	39.3	2.7	14-15 years	23.9	5.3	26.5	15.3
16-17 years	43.8	0.4	29.9	0.7	16-17 years	38.3	6.7	22.1	8.4
Disease duration	*n=307*		*n=411*		Disease duration	*n=271*		*n=340*	
<1 year	13.0	0.0	17.3	1.2	<1 year	11.4	1.1	11.5	7.1
1-2 years	26.7	1.0	24.3	1.5	1-2 years	19.9	8.1	15.3	8.8
3-4 years	23.5	0.0	23.1	1.0	3-4 years	17.0	5.2	17.9	6.5
5-6 years	13.4	0.3	11.9	0.5	5-6 years	12.6	2.2	8.2	4.1
>6 years	22.2	0.0	18.5	0.7	>6 years	18.5	4.1	12.4	8.2
**Nutritionists**	**Yes**	**No**	**Yes**	**No**	**Medical associations**	**Yes**	**No**	**Yes**	**No**
Age	*n=205*		*n=245*		Age	*n=200*		*n=220*	
12-13 years	20.0	6.3	14.7	13.5	12-13 years	20.5	6.0	17.3	12.7
14-15 years	22.4	6.3	22.4	18.4	14-15 years	25.5	5.5	25.0	15.0
16-17 years	34.6	10.2	20.0	11.0	16-17 years	33.0	9.5	18.2	11.8
Disease duration	*n=274*		*n=333*		Disease duration	*n=266*		*n=298*	
<1 year	11.3	1.1	9.3	9.0	<1 year	10.2	1.9	10.7	7.7
1-2 years	20.8	6.9	15.3	9.0	1-2 years	23.7	5.3	16.1	8.7
3-4 years	16.4	6.6	16.5	9.9	3-4 years	18.4	5.6	14.8	10.1
5-6 years	10.6	2.9	7.8	3.6	5-6 years	8.7	3.8	9.1	5.0
>6 years	15.3	8.0	7.2	12.3	>6 years	17.3	5.3	8.7	9.1
**Family, friends and acquaintances**	**Yes**	**No**	**Yes**	**No**	**Other patients**	**Yes**	**No**	**Yes**	**No**
Age	*n=209*		*n=253*		Age	*n=203*		*n=228*	
12-13 years	24.4	5.3	11.9	15.4	12-13 years	13.7	12.3	11.0	15.4
14-15 years	21.1	7.1	19.8	22.1	14-15 years	20.7	7.9	20.6	21.9
16-17 years	23.4	18.7	13.8	17.0	16-17 years	33.5	11.8	11.4	19.7
Disease duration	*n=278*		*n=341*		Disease duration	*n=270*		*n=307*	
<1 year	9.4	2.9	9.1	10.6	<1 year	7.0	4.4	10.1	8.8
1-2 years	20.1	9.0	9.7	14.4	1-2 years	18.9	9.3	12.7	12.1
3-4 years	15.5	6.8	11.7	12.0	3-4 years	13.3	9.6	9.4	15.0
5-6 years	10.1	4.3	5.3	8.2	5-6 years	11.5	2.6	5.9	6.2
>6 years	15.5	6.5	8.2	10.9	>6 years	16.7	6.7	8.5	11.4
**Pharmacists**	**Yes**	**No**	**Yes**	**No**	**Patient associations**	**Yes**	**No**	**Yes**	**No**
Age	*n=212*		*n=252*		Age	*n=189*		*n=230*	
12-13 years	15.1	11.3	11.9	16.7	12-13 years	13.8	12.7	15.7	11.7
14-15 years	18.9	10.0	20.6	20.2	14-15 years	22.2	7.4	27.8	14.8
16-17 years	31.1	13.7	15.5	15.1	16-17 years	28.0	15.8	18.7	11.3
Disease duration	*n=282*		*n=348*		Disease duration	*n=253*		*n=315*	
<1 year	8.9	3.9	7.5	11.5	<1 year	7.1	4.7	12.1	6.7
1-2 years	17.0	12.1	12.4	12.1	1-2 years	17.8	10.7	15.9	8.6
3-4 years	15.6	7.8	11.8	12.4	3-4 years	13.0	9.5	16.8	8.3
5-6 years	9.9	2.8	6.0	7.8	5-6 years	10.3	4.4	9.2	3.2
>6 years	13.5	8.5	8.3	10.3	>6 years	14.2	8.3	10.8	8.6

**Psychologist / Psychotherapists**	**Yes**	**No**	**Yes**	**No**	**Politics**	**Yes**	**No**	**Yes**	**No**
Age	*n*=201		*n*= 233		Age	*n*=191		*n*=217	
12-13 years	15.5	11.8	15.9	13.3	12-13 years	2.1	23.0	0.5	28.6
14-15 years	12.7	12.7	16.3	22.3	14-15 years	5.8	25.1	0.9	38.2
16-17 years	30.0	17.2	17.2	15.0	16-17 years	6.8	37.2	2.8	29.0
Disease duration	*n*=265		*n*=314		Disease duration	*n*=255		*n*=294	
<1 year	7.9	3.8	8.6	10.2	<1 year	1.6	10.0	0.7	17.7
1-2 years	17.0	11.0	12.7	10.5	1-2 years	3.6	25.2	0.3	23.7
3-4 years	15.1	8.7	13.4	11.5	3-4 years	4.0	18.8	1.4	23.7
5-6 years	9.4	4.9	8.3	4.8	5-6 years	2.8	11.2	0.3	11.2
>6 years	12.5	9.8	7.3	12.7	>6 years	2.8	20.0	0.7	20.1
**Self-help groups**	**Yes**	**No**	**Yes**	**No**	**Health insurance companies**	**Yes**	**No**	**Yes**	**No**
Age	*n*=180		*n*=204		Age	*n*=187		*n*=236	
12-13 years	8.9	18.3	11.8	17.2	12-13 years	9.6	17.1	12.3	17.8
14-15 years	14.4	12.2	16.2	24.5	14-15 years	14.4	15.0	12.3	27.1
16-17 years	26.1	20.0	13.2	17.2	16-17 years	21.4	22.5	11.9	18.6
Disease duration	*n*=243		*n*=277		Disease duration	*n*=252		*n*=320	
<1 year	4.5	7.4	9.7	10.5	<1 year	6.0	5.6	6.3	12.5
1-2 years	16.9	11.9	10.5	13.0	1-2 years	9.9	18.7	9.1	14.5
3-4 years	9.5	12.8	10.8	14.1	3-4 years	14.3	10.7	9.1	14.9
5-6 years	6.2	7.4	7.2	6.1	5-6 years	5.7	7.9	7.4	7.2
>6 years	9.9	13.6	6.5	11.6	>6 years	9.5	11.9	5.6	15.3
**Internet in general**	**Yes**	**No**	**Yes**	**No**	**Alternative medicine**	**Yes**	**No**	**Yes**	**No**
Age	*n*=202		*n*=268		Age	*n*=193		*n*=240	
12-13 years	8.9	17.3	11.2	14.6	12-13 years	12.4	14.0	6.3	20.5
14-15 years	12.4	17.8	22.8	21.6	14-15 years	9.8	19.7	10.8	28.8
16-17 years	19.8	23.8	14.2	15.7	16-17 years	17.6	26.4	10.8	20.8
Disease duration	*n*=265		*n*=380		Disease duration	*n*=257		*n*=328	
<1 year	5.3	7.6	8.4	13.7	<1 year	6.6	4.3	4.9	14.0
1-2 years	10.9	17.7	11.6	11.6	1-2 years	10.9	18.3	6.4	16.8
3-4 years	8.7	14.3	10.8	12.6	3-4 years	9.7	14.8	7.0	18.3
5-6 years	5.7	8.3	5.8	6.6	5-6 years	3.5	10.5	4.3	8.2
>6 years	10.6	10.9	10.0	8.9	>6 years	9.3	12.1	6.4	13.7
**Pharmaceutical industry**	**Yes**	**No**	**Yes**	**No**	**Special internet forums, chatrooms**	**Yes**	**No**	**Yes**	**No**
Age	*n*=186		*n*=222		Age	*n*=195		*n*=229	
12-13 years	6.5	21.5	6.8	23.0	12-13 years	6.2	18.5	7.4	21.4
14-15 years	9.7	18.8	9.5	30.6	14-15 years	11.3	18.5	12.2	27.5
16-17 years	16.1	27.4	5.4	24.8	16-17 years	16.9	28.7	8.3	23.1
Disease duration	*n*=260		*n*=310		Disease duration	*n*=254		*n*=310	
<1 year	1.9	11.9	1.9	17.1	<1 year	3.9	8.3	6.5	11.9
1-2 years	5.4	22.3	5.8	16.8	1-2 years	8.7	20.9	5.8	17.7
3-4 years	5.4	18.5	6.5	17.7	3-4 years	6.7	15.0	6.5	18.7
5-6 years	0.8	12.3	3.5	11.9	5-6 years	4.7	9.8	6.1	7.1
>6 years	3.9	17.7	3.5	15.2	>6 years	7.9	14.1	5.5	14.2
**Transition programs**	**Yes**	**No**	**Yes**	**No**	**Television, Consumer programs**	**Yes**	**No**	**Yes**	**No**
Age	*n*=165		*n*=164		Age	*n*=196		*n*=245	

12-13 years	5.5	21.8	9.1	22.6	12-13 years	4.6	21.4	9.0	18.4
14-15 years	7.3	21.8	10.4	29.3	14-15 years	9.7	19.9	11.0	31.0
16-17 years	17.6	26.1	8.5	20.1	16-17 years	13.3	31.1	8.2	22.4
Disease duration	*n*=223		*n*=216		Disease duration	*n*=255		*n*=328	
<1 year	5.4	6.3	5.6	14.4	<1 year	2.8	9.0	4.3	13.7
1-2 years	6.3	21.1	3.7	18.1	1-2 years	6.3	22.0	5.2	18.6
3-4 years	7.2	15.7	7.4	18.5	3-4 years	9.4	13.7	7.3	17.4
5-6 years	4.5	9.9	3.7	7.9	5-6 years	3.1	11.4	4.6	8.8
>6 years	8.1	15.7	5.1	15.7	>6 years	6.7	15.7	3.7	16.5
